# *Staphylococcus*-associated marginal keratitis secondary to pterygium surgery: a case report

**DOI:** 10.1186/s12886-021-01914-6

**Published:** 2021-03-31

**Authors:** Xin Liu, Xiancheng Ye, Yanlong Bi

**Affiliations:** 1 Department of Ophthalmology, Tongji Hospital Affiliated with Tongji University School of Medicine, No. 389, Xincun Road, Putuo District, Shanghai, 200065 China; 2 Department of Ophthalmology, Guizhou Provincial People’s Hospital, Guiyang, Guizhou China

**Keywords:** Pterygium excision, *Staphylococcus aureus*, Marginal keratitis, Blepharoconjunctivitis, Case report

## Abstract

**Background:**

*Staphylococcus*-associated marginal keratitis is an immune-mediated corneal disorder mainly secondary to chronic blepharoconjunctivitis. We report a rare case of *Staphylococcus*-associated marginal keratitis following pterygium excision. To the best of our knowledge, none of the previous literature has described such an acute complication after pterygium surgery.

**Case presentation:**

We report a case of a 50-year-old woman who suffered from pterygium in the left eye and underwent pterygium surgery. After surgery, slit-lamp examination showed an incomplete ring-shaped creamy white infiltrate. Corneal pathogenic microbial detection was negative. *Staphylococcus aureus* was found on the upper eyelid margin of the affected eye. Therefore, she was clinically diagnosed with *Staphylococcus*-associated marginal keratitis. The infiltrate was gradually absorbed after steroids, topical antibiotics, and lubricant eye drops were administered. After 2 years of follow-up, neither corneal infiltrate nor pterygium recurrence was observed.

**Conclusion:**

*Staphylococcus*-associated marginal keratitis is an immune reaction mainly secondary to chronic blepharoconjunctivitis, which usually activates an antigen-antibody reaction with complementary activation and neutrophil infiltration in patients sensitized to staphylococcal antigens. Early detection and treatment is of great importance. Topical steroids are effective and should be initiated early once pathogenic microbial infections are excluded. Although chronic staphylococcal blepharoconjunctivitis is a common disease, ophthalmologists should pay more attention to it to avoid potential complications.

## Background

Pterygium is a common ocular surface disease that is usually benign, and surgery is the only curative treatment. Reported complications include keratitis, scleral ulceration, necrotizing scleritis, perforation, iridocyclitis, cataract formation, glaucoma, and scleral calcification [1]. *Staphylococcus*-associated marginal keratitis, also called catarrhal ulcers, was first recorded by Thygeson in 1946 and is usually regarded as a complication of chronic staphylococcal blepharoconjunctivitis [2]. *Staphylococcus aureus (S. aureus)* plays an important role in the onset of catarrhal ulcers, which usually activate an antigen-antibody reaction with complementary activation and neutrophil infiltration in patients sensitized to staphylococcal antigens [3, 4]. To the best of our knowledge, this is the first report of *Staphylococcus*-associated marginal keratitis following pterygium surgery.

## Case presentation

A 50-year-old woman suffering from nasal pterygium in the left eye came to our clinic and sought pterygium surgery. The patient had no specific medical or surgical history. She had best corrected visual acuity (BCVA) of 20/20 in both eyes. There were no obvious abnormalities on the external eye examination, slit-lamp examination or fundus examination. Then, the patient underwent successful pterygium excision with conjunctival autografts in our clinic.

**Fig. 1:**
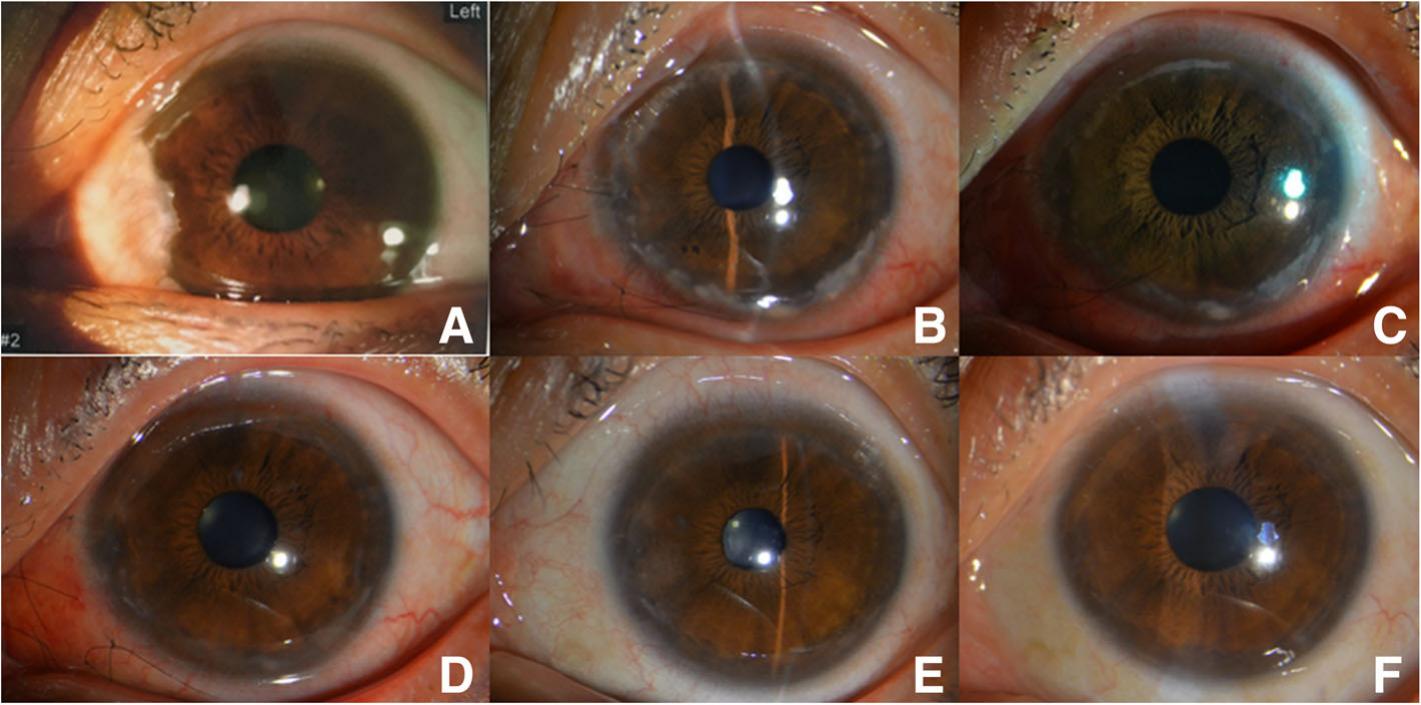
Slit-lamp examination. **a** Preoperative slit-lamp photography of the left eye. **b** Slit-lamp photography of the left eye 1 day after pterygium excision with conjunctival autografts. Note the incomplete ring-shaped creamy white infiltrate with an intact corneal epithelium over the infiltrate that left a clear space between the infiltrate and limbus. **c** Two days after treatment. **d** Eight days after treatment. Note the infiltrate being gradually absorbed. **e** Three months after treatment. Note that there was a mild stromal haze peripherally. **f** Fifteen months after treatment. Note that the cornea is clear, and neither corneal infiltration nor pterygium recurrence is seen

**Fig. 2:**
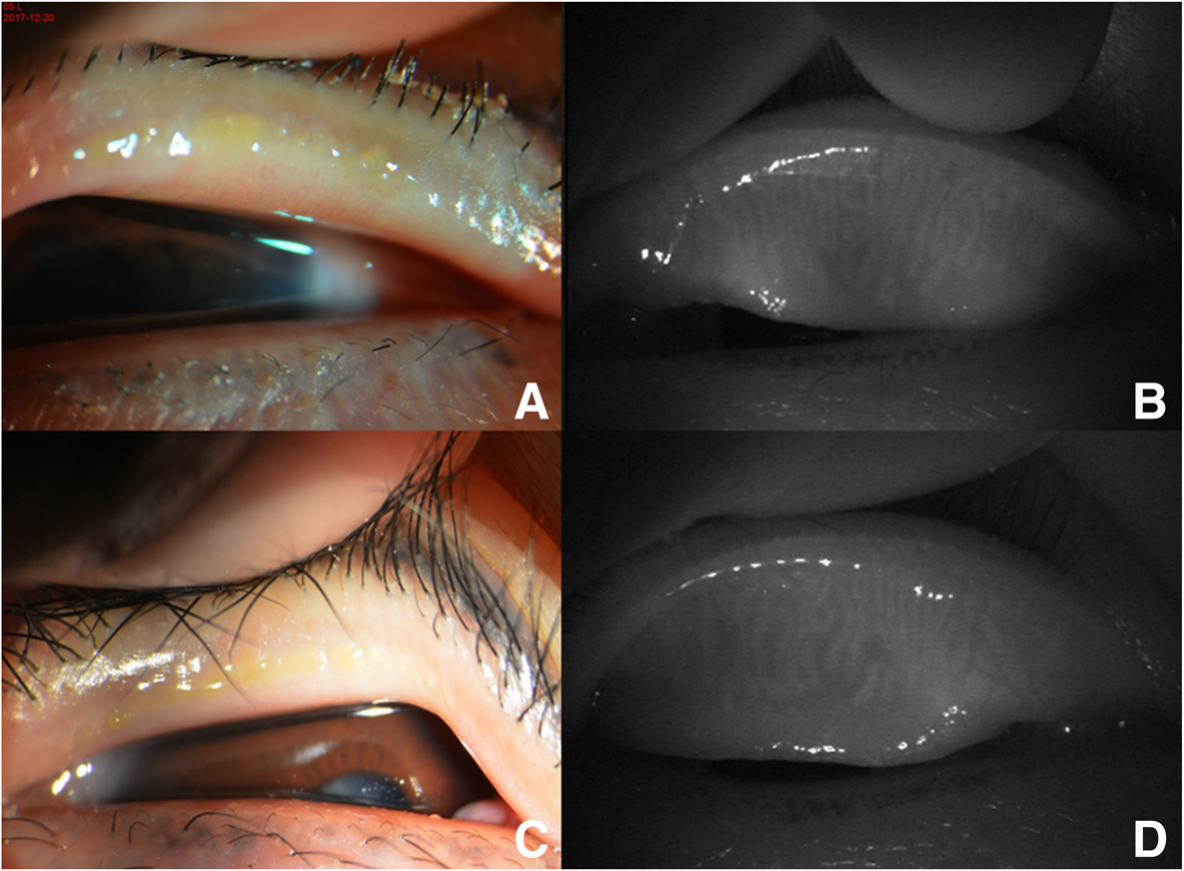
**S**lit-lamp and meibomian gland infrared photography. **a** A slit lamp showed meibomian gland obstruction and yellow discharge of the left upper eyelid. **b** Meibomian gland infrared photography showed partial meibomian gland loss of the left upper eyelid. **c** A slit lamp showed meibomian gland obstruction and yellow discharge from the right upper eyelid. **d** Meibomian gland infrared photography showed partial meibomian gland loss of the right upper eyelid

On the first day after surgery, the patient was in moderate pain with tearing and photophobia. Her BCVA was 20/20. Slit-lamp examination showed an incomplete ring-shaped creamy white infiltrate with an intact corneal epithelium over the infiltrate that left a clear space between the infiltrate and limbus (Fig. 1) and was associated with mild meibomian gland obstruction (Fig. 2), conjunctival congestion, no obvious ocular discharge, quiet anterior chamber, and a normal fundus examination. Corneal scraping from the affected eye, the conjunctival sac discharge and the upper lid margin discharge in both eyes were sent for Gram and Giemsa staining and pathogenic microbial detection. The upper lid margin in both eyes was positive for *S. aureus*, but it was negative in the corneal scraping and conjunctival sac. Meibomian gland infrared photography showed partial meibomian gland loss (Fig. 2). The examination results included hematology analysis, erythrocyte sedimentation rate (ESR), C-reactive protein (CRP), rheumatoid factor, antinuclear antibody, antineutrophil cytoplasmic antibodies, TORCH infections, and T-SPOT. TB assays were all negative. Combining the above results, we clinically diagnosed the patient with S*taphylococcus*-associated marginal keratitis. Daily eyelid hygiene was advised, and 1% prednisolone acetate, 0.3% levofloxacin and 0.1% sodium hyaluronate eye drops were administered 4 times a day under close observation. The infiltrate was gradually absorbed after initiation of the medication. One week later, the infiltrate was nearly absorbed and left only a mild stromal haze. After 2 years of follow-up, neither corneal infiltration nor pterygium recurrence was observed. The patient was satisfied with the treatment effect.

## Discussion and conclusions

Pterygium surgery is usually thought to have a low rate of infectious complications. Nevertheless, infectious complications could unexpectedly occur a few days or even a few years after surgery. Mohammad et al. found an incidence of 2/1000 for microbial keratitis after pterygium surgery [5].

*Staphylococcus*-associated marginal keratitis is an immune-mediated corneal disorder secondary to longstanding staphylococcal blepharoconjunctivitis, which typically manifests as a gray-white, ring-shaped infiltrate, which is always located approximately 1 mm inside the limbus, with a characteristic clear zone of cornea between the infiltrate and the limbus, and there is little tendency to spread centrally or peripherally [6]. The infiltrate appears first and is followed by fluorescein staining as ulceration develops [7].

To the best of our knowledge, *Staphylococcus*-associated marginal keratitis could occur in an acute, subacute or chronic onset. In some cases, the disease can be recurrent with longstanding staphylococcal blepharoconjunctivitis. There is no doubt that we should distinguish this disease from direct infectious keratitis. Microbial keratitis after pterygium surgery usually occurs in the operative site and is accompanied by severe pain, conjunctival congestion, and ocular discharge. The gold standard of diagnosis is the detection of pathogens and the effectiveness of antibiotic eye drops. Therefore, combined with the characteristics of our case, we clinically diagnosed this patient with S*taphylococcus*-associated marginal keratitis.

In 1946, Thygeson isolated *S. aureus* from the lids of 133 of 156 patients with catarrhal keratoconjunctivitis [2]. Hogan et al. noted that lid cultures usually grew *S. aureus* in marginal keratitis, but their corneal cultures were negative for bacteria [8]. This reveals that the lesion is probably not triggered by direct infection of the cornea but by a hypersensitivity reaction, in which antigen-antibody reactions with complement activation and neutrophil infiltration in patients are sensitized to staphylococcal antigens [9]. Ueta et al. suggested that the *S. aureus* present in the lid margin, rather than the conjunctival sac, was more important for the development of *Staphylococcus*-associated marginal keratitis [10]. Rao et al. proposed that increased expression of meibomian secretions and bacterial toxins after manipulation of the lid margin could trigger immunological reactions with infiltration. If this theory is true, there should be a more marginal infiltrate after ocular surgery, since lid inflammation is common [11].

Interestingly, Ueta et al. found the same clone of *S. aureus* in the margin of the unaffected eye [10]. In the case presented herein, the same clone of *S. aureus* was also found in the upper lid margin and it was negative in the conjunctival sac of the unaffected eye. This revealed that in addition to the existence of *S. aureus* on the lid margin, we need to assume that other factors, such as the patients’ high sensitivity to *S. aureus*, chemical or physical stimulation, differences in the number of bacteria between the affected eye and unaffected eye, etc., may be trigger factors for the initiation of *Staphylococcus*-associated marginal keratitis.

In summary, the incidence of infectious keratitis following pterygium excision is low, and ophthalmologists should be aware of potential infectious complications that can have dramatic outcomes. *Staphylococcus*-associated marginal keratitis is a rare surgical complication after pterygium excision, and *S. aureus* colonization of the lid margin may be one of the most important causes. Topical steroids have a significant effect and should be initiated as soon as possible to minimize the lesions once direct infections by pathogenic microorganisms are excluded. In most cases, the patients had a favorable prognosis. Since there are many people with longstanding staphylococcal blepharoconjunctivitis, it is clear that only a few of them have developed marginal keratitis. Therefore, the pathogenesis of *Staphylococcus*-associated marginal keratitis deserves further study.

## Data Availability

Not applicable.

## Abbreviations

S aureus: *Staphylococcus aureus*
BCVA: Best corrected visual acuity
ESR: Erythrocyte sedimentation rate
CRP: C-reative protein
